# Health care providers’ perspective on using family history in the prevention of type 2 diabetes: a qualitative study including different disciplines

**DOI:** 10.1186/1471-2296-14-31

**Published:** 2013-03-07

**Authors:** Suzanne CM van Esch, Wieke H Heideman, Wilmy Cleijne, Martina C Cornel, Frank J Snoek

**Affiliations:** 1 Department of Medical Psychology, VU University Medical Center, Van der Boechorststraat 7, Amsterdam, The Netherlands; 2 EMGO Institute for Health and Care Research, VU University Medical Center, Van der Boechorststraat 7, Amsterdam, The Netherlands; 3 Department of Clinical Genetics, VU University Medical Center, Van der Boechorststraat 7, Amsterdam, The Netherlands

**Keywords:** Type 2 diabetes, Family history, Primary prevention, Family health, Health promotion, Family communication, Interview study

## Abstract

**Background:**

Family history (FH) is considered an important factor to detect individuals at increased risk developing type 2 diabetes (T2D). Moreover, FH information could be used to personalise risk messages, which are assumed to increase risk-reducing behaviours. In this study, we aimed to explore Dutch health care professionals’ attitudes regarding current or future uptake of a more extensive use of FH information and the family system in diabetes prevention.

**Methods:**

Semi-structured interviews were conducted with nineteen health care professionals from six general practices and four outpatient diabetes clinics. The use of FH information in opportunistic screening for T2D was explored, as well as the usability of a direct versus patient-mediated targeting strategy to reach persons with a FH of T2D. Three researchers analysed the interview transcripts separately.

**Results:**

Dutch health care professionals considered FH an important risk factor in opportunistic screening for T2D. However, none of them used FH to promote risk-reducing behaviours. Directly targeting and educating patients known to have a FH of T2D was desirable for most primary care professionals, but not considered feasible. Findings indicated that FH information was not systematically gathered in primary care settings and electronic medical records were not equipped to retrieve persons with T2D running in their family. The idea of asking patients to pass on risk and preventive information was new to all interviewees, but was considered an acceptable strategy to reach persons with a FH of diabetes. Nevertheless, there were concerns about the accuracy of the messages delivered by the patients to their relatives. Practical barriers with regard to time, expertise, and financial reimbursement were also mentioned.

**Conclusions:**

There is great interest among healthcare professionals in primary as well as secondary care about the use of FH to prevent T2D, but there are significant barriers against such use. The removal of these barriers would depend on evidence showing the cost-effectiveness of FH-based strategies designed to prevent T2D.

## Background

Type 2 diabetes (T2D) is a serious chronic disease causing considerable burden on patients as well as health care systems [1]. The rapidly increasing prevalence of T2D warrants major efforts to explore effective tools and strategies to detect, inform and motivate individuals at high risk to engage in preventive actions [2].

T2D is best described as a multi factorial disease, which means disease onset is triggered by the interaction of multiple genes and environmental factors [3]. Research has convincingly demonstrated that T2D is highly prevalent in some families and a positive family history predicts the development of T2D, even after adjustment for common risk factors [4-6]. Consequently, a family history (FH) of T2D is seen as a valuable tool in preventive activities [7]. FH information can help to detect persons at increased risk developing T2D: the chance of developing diabetes is two-to-five times higher for people with a FH of the disease [6,8,9]. Moreover, evidence suggests that preventive messages tailored to a person’s FH can increase risk awareness and risk-reducing behaviours [10-12]. Yet, interventions using FH information to promote a healthy lifestyle seem to be scarce [13].

In most Western countries, the use of opportunistic screening targeting patients at risk for T2D is encouraged [14]. In combination with other risk factors, FH is recognized as an important element in the risk stratification procedure. Directly targeting and educating people known to have T2D running in their family, however, is not current in clinical practice [11,12,15]. Yet, there is an increasing need for structured preventive activities linked to primary care [16]. A targeting approach that might be worthwhile to explore in this context is patient-mediated cascading, as is used in screening for familial hypercholesterolemia [17]. This means that relatives are reached via the index patient, who informs them about increased familial susceptibility to a disease and the preventive options. Research already has indicated that the majority of patients with T2D seems willing to disseminate risk and preventive information in their family [18,19]. Adult offspring generally appears receptive to be informed about reducing their diabetes risk via the family system [20].

In this study, semi-structured interviews with Dutch health care professionals were conducted to investigate opinions, attitudes and practices with regard to the current and future use of FH in preventive consultations. Ideas regarding two potential strategies to reach persons with T2D running in their family were explored: 1) directly targeting patients known to have a FH of T2D and 2) a patient-mediated approach, asking patients with T2D to pass on risk information to first-degree and second-degree relatives. After all, increased T2D risk is present in first-degree as well as second-degree relatives [8,21]. Moreover, although preventive activities usually are carried out in primary health care, we considered opinions of secondary care professionals also of relevance in this study. Patients receiving diabetes treatment in secondary care visit outpatient clinics regularly, which might provide opportunities to discuss the familial character of the disease. Findings may provide insight in the uptake of FH as a tool in T2D prevention and the conditions that are needed to apply family-based strategies in routine care.

## Methods

### Design and participants

Between February and April 2010, two researchers (SvE and WH) conducted nineteen semi-structured interviews with Dutch health care professionals. A purposive sampling strategy was used, aiming to achieve maximum variation in the characteristics of the included affiliations and professionals [22]. General practices were recruited via the regional network of the Academic General Practice of the VU University Medical Center. Six practices in three cities in the urban area of Amsterdam were willing to participate. Interviews were conducted with five general practitioners (GPs) and six practice nurses (PNs). The term ‘general practice’ is commonly used in the Dutch health care system and is considered synonymous to ‘family medicine’ and ‘family practice’.

Diabetes specialists (DSs) and diabetes nurses (DNs) in secondary care were approached via contacts of the authors in four outpatient diabetes clinics in Amsterdam. Interviews were conducted with five DSs and three DNs. The study sample included professionals of both sexes and varying years of clinical experience (see Table 1). All interviewees were of Dutch origin. Affiliations varied in size and characteristics of patient populations (see Table 2). All interviewees gave informed consent prior to the interview. The VU University Medical Center Ethics Committee approved the study.

**Table 1:** **Distribution of interviewees according to gender and years of clinical experience**^**#**^

	**Primary care**		**Secondary care**	
	**GPs**	**PNs**	**DSs**	**DNs**
Included practices/outpatient clinics	6		4	
Number of interviewees^§^	5	6	5	3
Gender				
Male	3	-	4	1
Female	2	6	1	2
Clinical experience				
0-10 years	-	6	2	2
11-20 years	2	-	2	-
21-30 years	1	-	1	1
> 31 years	2	-	-	-

Notes: GPs=General practitioners, PNs=Practice Nurses, DSs=Diabetes Specialists, DNs=Diabetes Nurses.

^#^ All interviewees were from Dutch origin.

^§^ In one general practice, the GP and PN were interviewed at the same time. In another general practice, two PNs were interviewed at the same time. One PN worked in two general practices that were both included in the study.

**Table 2:** **Distribution of the general practices (N=6) and outpatient clinics (N=4) according to key characteristics of their patients**^‡^

	**Primary care**	**Secondary care**
Social economic status		
Low	3	3
Low and average	1	-
Average	1	-
Average and high	1	1
Ethnic backgrounds		
Majority of Dutch origin	4	1
Majority of non-Dutch origin	2	3
Age distribution		
Normal distribution	4	4
> 65 years overrepresented	2	-

^‡^Professionals were asked to define their patient population according to socioeconomic status (SES), ethnic backgrounds and age distribution.

### Interview guide

The interviews were semi-structured and based on a topic guide (see below) that was pilot tested in two interviews. In the first part of the interview, interviewees were asked to describe current practice with regard to the use of (opportunistic) screening for T2D, assessment and registration of FH and structured education targeting patients at risk developing T2D. Individual opinions with regard to bringing up FH as a topic of conversation in preventive consultations were explored. Next, two potential strategies to reach relatives of T2D patients were discussed: 1) directly targeting patients known to have a FH of diabetes and 2) indirectly targeting, i.e. asking patients to pass on information to first-degree and second-degree relatives. Considerable flexibility during the interviews allowed interviewees to discuss and elaborate on issues that were most important to them. Each interview lasted about half an hour and took place in the participants’ work environment.

The interview guide included the following topics (* indicated topics are only discussed with primary care professionals).

#### Introducing the interview

“*With the increasing incidence, primary prevention of type 2 diabetes is of key importance. Clinical and public health efforts are generated to assist in reducing the burden of diabetes in the population. In this interview, we aim to explore the current and future uptake of proactive patient education about familial susceptibility to type 2 diabetes*”.

#### Mapping current practice

• Are patients at risk developing type 2 diabetes systematically screened in this general practice?*

○ In case of opportunistic screening: which risk factors are assessed?

○ Can you describe the practical implications of the screening process?

• Is family history of type 2 diabetes systematically assessed (if yes, how registered)?

• Is education about risk factors and preventive options systematically offered when patients are at risk developing type 2 diabetes (but not yet diagnosed)?*

○ Which risk factors are emphasized?

○ To which extent is family history discussed?

#### Exploring perceptions and attitudes

• What is your opinion about using family history as a topic of conversation to promote health protective behaviour?*

○ Do you use family history to personalize preventive messages?

○ Do you think it will be effective to promote healthy behaviour?

• What is your opinion about proactively targeting patients with a family history of type 2 diabetes to educate them about preventive options?*

○ Do you think it is effective (e.g., with regard to patient empowerment/responsibility/ privacy)?

○ Do you think it is feasible?

• What is your opinion about asking patients to deliver diabetes risk and preventive messages in their family (first-degree as well as second-degree relatives)? Elaborate on:

○ Patients’ willingness

○ Potential effect on relatives

○ Feasibility

○ Familial and/or cultural aspects

• What would be needed to implement family-based strategies in diabetes prevention?

• Are you familiar with the information that is provided by mass media campaigns and websites aiming to raise public awareness about familial susceptibility to type 2 diabetes?

○ Do you use this information and/or refer patients to these websites?

### Data analysis

All interviews were digitally recorded, transcribed verbatim (WC) and checked for errors (SvE and WH). Qualitative data indexing software (ATLAS.ti 5.2) was used for data coding and retrieval. The transcripts were analysed using thematic content-analytical techniques. Main codes were established for the core questions in the interview guide; sub-codes were inductively formulated to identify emerging sub-themes. Two investigators (SvE and WC, or WH and WC) independently coded each transcript. Ambiguities in the final code-lists were discussed until consensus was reached. Subsequently, (sub-)codes were grouped in thematic matrices and similarities, variations and patterns amongst the professional groups were summarized. Main findings were discussed with all members of the study group. The quotations that follow were chosen to reflect a range of both consensual and dissenting views. Identification numbers are placed between square brackets and include participants’ professional background (GP, NP, DS or DN) and the number that was assigned to the practice/clinic.

## Results

### Mapping current practice

All included GPs work in accordance with the Dutch guideline for diabetes treatment, which include opportunistic screening for T2D [23]. One GP participated in a trial to implement the screening protocol for cardio-metabolic prevention, targeting all patients >55 years old [16,24]. All interviewees perceive FH (specifically in first-degree relatives) as important factor in the risk stratification procedure. However, according to the interviewees in primary care, the assessment of FH information is not standardized; a person’s FH of diabetes is inquired the moment it is thought to be of relevance. Professionals vary in asking about second-degree relatives with T2D. In diabetes outpatient clinics, FH is systematically assessed during the first consultation. Both in primary and secondary care, FH information is registered in electronic medical records (EMRs), but not with a retrievable code. When patients are diagnosed with (pre)diabetes, they are regularly monitored and receive education about T2D risk factors and lifestyle modifications to prevent diabetes complications (secondary prevention).

### Using family history information in preventive actions

Data revealed that the extent to which the multifactorial aetiology of T2D is explained varied between professionals: *‘It depends on the patient, whether (s)he is interested. But I try to explain that some people are more at risk than others.’* [GP4]. Some GPs and PNs do not emphasize the role of FH as a risk factor, as it is not a factor that can be changed: *‘We think monitoring weight and blood glucose levels in this population is most effective. We don’t emphasize family history.’* [GP3] *‘Understanding the balance between food consumption and energy expenditure, that’s what counts.’* [GP5].

None of the interviewees used FH information to promote health-protective behaviour. The clarification of what FH could mean to a person by professionals seems to be minimal. Professionals could not bring up absolute or relative risk estimates of developing T2D in persons with a FH. Nevertheless, they agreed that personal perceptions about diabetes running in the family could be discussed more thoroughly and knowledge about familial susceptibility to diabetes could be improved: *‘There is a lot of ignorance. […] People don’t recognize diabetes symptoms, despite the –sometimes high- diabetes prevalence in their family.’* [GP4]. The idea of using FH information to motivate risk-reducing behaviour was new to all interviewees, but it was acknowledged that for some relatives, personalized risk messages could be a cue to action: *‘I think, targeting family members could be effective. However, I think you should reach them in a neutral and thoughtful manner. People don’t want you to interfere with their personal life too much.’* [GP2].

All primary care professionals reported to be interested in new strategies and tools to inform people about the importance of a healthy lifestyle. Interestingly, with exception of one PN, none of the interviewees had paid attention to or used the information provided by renowned Dutch health organizations. Between 2009 and 2013, diverse mass medial campaigns and an informative website were launched [25], providing a diabetes risk test that generates personalized preventive information [26].

### Directly targeting patients at familial risk developing type 2 diabetes

Most GPs and PNs indicated that directly targeting and educating populations at risk, including persons with a FH, would be desirable and worthwhile: *‘We plan to set up more preventive activities targeting patients with an extensive family history of cardiovascular disease and type 2 diabetes.’* [GP4]. However, they foresee practical problems; lack of time, finance and organizational barriers were reported: ‘*What we need is a continuing approach. Our PNs are trained to provide patient education and motivate patients in the process of behaviour change. […] We could organize and facilitate a structured programme, on condition that financial resources are available.’* [GP2]. Most importantly, however, directly targeting patients with a FH is not possible because EMRs are not equipped to retrieve persons with a FH: ‘*The most important barrier is to identify and reach patients with an extensive family history.’* [PN6b].

### Asking patients to pass on risk and preventive messages in their family

The idea of asking patients to inform relatives about familial susceptibility to T2D appeared to be new to all interviewees. During the interviews, the professionals became more and more interested in this potential approach to reach relatives at risk: *‘When you think about prevention, you have to reach as much people as possible. I do not disapprove this kind of targeting approach.’* [PN4&5]. Interviewees referred to patients who bring up inheritance and concern about the future health of their relatives themselves. They commended on the possibility of contacting otherwise unreachable healthy individuals and thought that a specific group of patients seems willing to disseminate information in their family: *‘Patients who adequately handle their disease will be motivated to participate. Other patients are into denial and/or struggling with their disease. You can’t ask these patients to deliver diabetes risk messages in their family.’* [PN6a].

However, for some GPs it was difficult to think about targeting a population that does not necessarily include their own patients: *‘I think it is difficult to manage, sometimes I ask about relatives, but most relatives are not registered as a patient in our practice.’* [GP1]. Moreover, besides a lack of time during their consultations, they indicated that they would need expertise and skills to guide and educate patients who are willing to serve as a messenger in their family.

### Family-based diabetes prevention in secondary care

Most interviewees in secondary care do not think they should have an active role in the primary prevention of T2D, however, they are open to the idea of informing patients and their relatives about familial susceptibility to T2D: ‘*Indeed, we talk about family history. When patients or relatives ask about it, I inform them about the importance of a healthy lifestyle and advice relatives to consult their GP for a yearly check-up.’* [DS2]. One DS realized that in other situations, they have a more active role with regard to prevention in families at high risk developing a disease: ‘*We always inform patients about familial susceptibility in case of monogenetic disorders. The problem with T2D is its multifactorial aetiology; the message is not clear and more difficult to explain.’* [DS1].

One DN thinks every health care professional should be concerned about a population at risk, but emphasized that there are little opportunities to act upon that in secondary care. Other professionals were interested in the idea of initiating conversations in families at risk: *‘I think patients can tell from their own experience what it’s like to have the disease […] Most of my patients have adult offspring. That would be a good target population.’* [DS4]. They emphasized the importance of repeating health-protective messages: *‘Repetition is important in health education. […] It seems a good idea that people hear the same message over and over again: from public health communications, in general practice, from dieticians and from us.’* [DS1]. Another professional, however, stated that a patient-mediated targeting approach is not appropriate in secondary care: ‘*Some patients in secondary care are quite sick. You can’t ask them to inform their relatives.’* [DN2].

### Perceived barriers regarding a patient-mediated targeting approach

Notwithstanding the interest and enthusiasm of most interviewees, some questioned the feasibility and benefits of a patient-mediated approach in diabetes prevention: ‘*Patients don’t want to deliver bad news and relatives don’t want to receive such messages*.’ [DS3a]. They doubted whether patients would be able to pass on accurate messages and whether relatives will be alarmed: ‘*Will the messages be delivered? To be honest, considering our patient population, I suppose a substantial amount will not.’* [PN4&5]. As T2D is a lifestyle related disease, many patients would not fulfil a role model with regard to health behaviour: *‘I think the most important factor is how patients experience and cope with their disease and how visible it is for relatives.’* [PN1]. Interestingly, nurses (PNs and DNs) seemed to be more hesitative than medical professionals (GPs and DSs).

Different professionals mentioned strong family bonding in ethnic minority families as a potential advantageous factor: *‘Family tights seems to be stronger in immigrant families.’* [DS3]. Conversely, other professionals emphasize cultural and linguistic barriers. Some GPs and PNs do not expect benefits from illuminating the familial character of T2D in ethnic minority groups because of differences in perceived controllability with regard to health and illness, causal attributions, generational conflicts and low literacy: *‘The illness burden of first generation migrants might not impress the younger generations. These youngsters do not identify with their parents as far as health-related issues are concerned.’* [GP5]. Asking non-Dutch patients to pass on information seems not feasible according to these professionals: *‘It’s the other way around. Those children are used to translate during consultations and provide their parents with health information. They won’t listen to their parents and will search for information themselves when they need it.’* [GP4].

Moreover, the younger generation in general would not be admissible to risk messages via the family system: *‘I question whether it’s effective. Younger offspring is not concerned with future health risks.’* [GP1] *‘Do children listen to their parents? […] I think a person will be interested the moment (s)he is confronted with the problem.’* [DS3a].

## Discussion

Findings in this study indicated that Dutch health care professionals seem to be interested in a more extensive use of FH information, even though they were unacquainted with the idea of discussing a person’s understanding of familial risk to promote and motivate health-protective behaviour. However, the effectiveness of illuminating FH was questioned, especially with regard to patients of non-Dutch descent and younger generations. Studies have indicated that targeted diabetes education actually does increase the recognition of diabetes risk, screening possibilities, perceived personal control and the need of healthy behaviour in persons with a FH of T2D [10-12,27,28]. Moreover, relatives who were informed via the family system perceived themselves at increased risk developing diabetes [20,29]. Nevertheless, professionals are right in stating that the effect of using FH in preventive communications targeting specific populations remains undetermined [10-12]. We only know that in families with different ethnic backgrounds (e.g., South-Asian, Middle-Eastern), family communication about T2D is not a taboo and patients seem willing to pass on risk and preventive information in their family [18,30].

Generally, the study findings lend support for the adoption of direct as well as indirect (patient-mediated) strategies targeting persons with T2D running in their family. Both methods seem effective in other disease areas [17]. In Dutch primary care practice, a direct and active invitation of the GP increased the screening uptake of participants for a lifestyle intervention on T2D risk reduction [16,31]. According to some interviewees, however, structurally targeting individuals with a FH of T2D was not considered feasible. Besides practical barriers with regard to time, expertise and financial reimbursement, they indicated that FH is not systematically registered and EMRs are not equipped to retrieve persons with a FH of T2D. This problem was reported in earlier research [15,32]. In the future, the development of tools to collect standardized FH that are compatible with EMRs may solve this obstacle [33]. Meanwhile, the implementation of a proactive disease prevention protocol linked to Dutch primary care may create opportunities to initiate conversations about FH more systematically [16,24]. A lifestyle intervention that uses FH to motivate relatives of T2D patients to maintain good health is currently being evaluated [34].

The idea of asking patients to pass on risk information was new to all interviewees, but was considered an acceptable strategy to reach persons with a FH. Nevertheless, quite a lot of professionals (especially nurses) were sceptical about the potential benefits of such a strategy. They doubted whether patients would be able to deliver accurate messages in their family. Research already has demonstrated that it would be advisable to provide patients with written information when they are asked to deliver risk and preventive messages in their family. Written information about familial hypercholesterolemia reduced patients’ hesitation and appeared to be helpful in the disclosure of family risk. In another study, information packages served as a cue to action for relatives and legitimated them to ask for a medical check-up [35].

A study in Japan has indicated that booklets with information about T2D risk and prevention, which were handed over by patients, worked effectively on attitudes and preventive behaviours in relatives. Yet, the reliability of patients as information deliverers appeared to be limited [36]. This latter finding underlines the doubts that some interviewees expressed in our study. Professionals’ concern that patients may fulfil a negative role model in their family was also indicated as a barrier in the disclosure of family risk by patients themselves [19]. Nevertheless, patients diagnosed with familial hypercholesterolemia seem to prefer a patient-mediated approach more than a direct targeting approach, as they consider it less threatening for relatives [17].

### Limitations

In this study, data were collected from a sample of diabetes care professionals in the Netherlands, representing most important disciplines in primary and secondary diabetes care. Interviews were conducted in an urbanised area, though there are no indications that this may limit the study’s generalizability to all diabetes professionals in the Netherlands. The Dutch health care system, however, might restrict generalizability to an international context. Moreover, the study sample could have had an impact on the results. Socio-demographic characteristics of the interviewees (for instance their age and national origin) could influence their view that non-Dutch and younger patients were least likely to respond to illuminating family history as a risk factor.

Finally, it should be noticed that the idea of asking patients to inform relatives about familial susceptibility to T2D was new to all interviewees. It would be interesting to explore opinions of professionals who have considered these issues more thoroughly, as (in)directly targeting relatives of index patients may raise ethical questions [17]. In addition, more insight is needed in cultural aspects regarding the disclosure of family risk, the effect of low health literacy and negative modelling in families at risk when utilizing family-based strategies in diabetes prevention.

## Conclusions

The results of this study suggest that health care professionals in primary, as well as secondary care are open to the idea of using FH in preventive activities. In Dutch primary care, the future implementation of a protocol for proactive prevention of non-communicable diseases [16,24] might provide opportunities to systematically discuss patients’ interpretation of familial susceptibility to a disease and potential effects on health-related behaviour. To start, professionals could use the (online) information that is made available by national public health initiatives to inform populations at risk developing T2D, including persons with a FH of the disease [37]. More importantly, however, for professionals to adopt family-based strategies in the prevention of T2D, convincing evidence is needed regarding the cost-effectiveness.

## Abbreviations

T2D: Type 2 diabetes; FH: Family history; GPs: General practitioners; PNs: Practice nurses; DSs: Diabetes specialists; DNs: Diabetes nurses.

## Competing interests

The authors declare that they have no competing interests.

## Authors’ contributions

SvE and WH developed the interview guide and conducted the interviews. WC transcribed the interviews verbatim and performed the analyses together with SvE and WH. SvE wrote the draft of the paper, which was discussed with WH. MC and FS participated in the design of the study, contributed to the interpretation of data, reviewed versions of the article and suggested revisions. All authors read and approved the final manuscript.

## Pre-publication history

The pre-publication history for this paper can be accessed here:

http://www.biomedcentral.com/1471-2296/14/31/prepub
